# Resistance to aspirin is increased by ST-elevation myocardial infarction and correlates with adenosine diphosphate levels

**DOI:** 10.1186/1477-9560-3-10

**Published:** 2005-07-26

**Authors:** Catharina Borna, Eduardo Lazarowski, Catharina van Heusden, Hans Öhlin, David Erlinge

**Affiliations:** 1 Department of Cardiology, Heart & Lung Division, Lund University Hospital, Sweden; 2 Department of Medicine, University of North Carolina, School of Medicine, Chapel Hill, USA

**Keywords:** aspirin, acute coronary syndromes, platelets, ADP

## Abstract

**Background:**

To be fully activated platelets are dependent on two positive feedback loops; the formation of thromboxane A_2 _by cyclooxygenase in the platelets and the release of ADP. We wanted to evaluate the effect of aspirin on platelet function in patients with acute coronary syndromes and we hypothesized that increased levels of ADP in patients with acute coronary syndromes could contribute to aspirin resistance.

**Methods:**

Platelet activity in 135 patients admitted for chest pain was assessed with PFA-100. An epinephrine-collagen cartridge (EPI-COLL) was used for the detection of aspirin resistance together with an ADP-collagen cartridge (ADP-COLL). ADP was measured with hplc from antecubital vein samples. Three subgroups were compared: chest pain with no sign of cardiac disease (NCD), NonST-elevation myocardial infarction (NSTEMI) and STEMI.

**Results:**

Platelet activation was increased for the STEMI group compared NCD. Aspirin resistance defined as <193 sec in EPI-COLL was 9.7 % in NCD, and increased to 26.0 % (n.s.) in NSTEMI and 83.3 % (p < 0.001) in STEMI. Chronic aspirin treatment significantly reduced platelet aggregation in NCD and NSTEMI, but it had no effect in STEMI. Plasma levels of ADP were markedly increased in STEMI (905 ± 721 nmol/l, p < 0.01), but not in NSTEMI (317 ± 245), compared to NCD (334 ± 271, mean ± SD). ADP levels correlated with increased platelet activity measured with ADP-COLL (r = -0.30, p < 0.05). Aspirin resistant patients (EPI-COLL < 193 sec) had higher ADP levels compared to aspirin responders (734 ± 807 vs. 282 ± 187 nmol/l, mean ± SD, p < 0.05).

**Conclusion:**

Platelets are activated and aspirin resistance is more frequent in STEMI, probably due to a general activation of platelets. ADP levels are increased in STEMI and correlates with platelet activation. Increased levels of ADP could be one reason for increased platelet activity and aspirin resistance.

## Background

To be fully activated platelets are dependent on two positive feedback loops; the formation of thromboxane A_2 _by cyclooxygenase in the platelets and the release of ADP from dense platelet granules. Thromboxane A_2 _and ADP then activates specific receptors on the extracellular side of the platelet membrane. Therapeutic intervention aimed at the first positive feedback loop by inhibiting cyclooxygenase with aspirin is highly efficient in reducing death and cardiovascular events by approximately 25% [1]. However, ADP may be even more important as evidenced by the CAPRIE-study, in which the ADP receptor antagonist clopidogrel was more beneficial than aspirin in reducing cardiovascular events [2]. Furthermore, the CURE and CREDO studies have established clopidogrel in combination with aspirin as a valuable treatment for acute coronary syndromes [3,4].

The platelet inhibitory effect of aspirin varies and aspirin resistance has been found in 9–45% of patients [5-7]. Little is known about the clinical consequences of aspirin resistance but lately two different studies indicate that aspirin resistance could be associated with an increased number of cardiovascular events [8,9].

Platelet activation is difficult to assess. Laboratory tests available are either not sufficiently reliable or rather complicated and therefore ineligible for clinical routine use. In this study we used a novel platelet function test, PFA-100. PFA-100 is an ex vivo assay of shear stress induced platelet adhesion and aggregation in whole blood. It simulates an injured blood vessel by a collagen-coated membrane together with either epinephrine or ADP. It has been found to be a sensitive test of aspirin resistance [6]. Aspirin resistance has previously been studied in healthy controls and in stable patients with a previous myocardial infarction.

In this study we wanted to evaluate the effect of aspirin on platelet function in patients with acute coronary syndromes. Furthermore, we hypothesized that increased levels of ADP in patients with acute coronary syndromes could contribute to aspirin resistance.

## Methods

### Patients

135 patients were enrolled from patients admitted for chest pain to the emergency ward, Lund University. Hospital between 2001–2003. Patients with chest pain within the last hour before admittance were eligible for inclusion. Patients were defined as aspirin users or patients not using aspirin the last three weeks (and not receiving aspirin during transport to hospital). The use of aspirin was defined as daily intake of aspirin for at least one week before admittance. Most patients were on aspirin 75 mg once daily, but a few (< 10%) were on 320 mg once daily. Exclusion criteria were: ingestion of clopidogrel, dipyridamole, nonsteroidal antiinflammatory drugs, heparin, low molecular heparin, warfarin, receiving bolus dose of aspirin on their way to hospital, platelet count <140 × 10^9^/l, hemoglobin <90 g/l, renal failure (creatinine >140 μmol/l) or hemolysis in blood sample. The Human Ethics Committee of Lund University approved the project. All participants gave informed written consent before enrolment.

Based on the diagnosis at discharge, three prespecified subgroups were compared: chest pain with no sign of cardiac disease (NCD), Non ST-elevation myocardial infarction (NSTEMI) and STEMI. The NCD group presented no recent ECG changes and normal values of TNT. Exercise test where appropriate before discharge were negative. A minor number of patients in the NSTEMI and STEMI group were on beta-blocker, ACE-inhibitors and Ca-channel blockers. However, the groups were to small for subgroup analysis.

### PFA-100 system testing

The PFA-100 system has been described in detail by Kundu and co-workers [10]. The PFA-100 uses a disposable test cartridge that simulates an injured blood vessel. The PFA-100 simulates primary haemostasis by flowing whole blood at a high shear rate through an aperture (147 μm diameter) cut into a collagen-coated membrane coated with either ADP (50 μg) or epinephrine (10 μg), where it comes into contact with the membrane surface and aggregate. A platelet plug forms, with occlusion of the aperture and cessation of blood flow. The closure time reflects platelet function in the sample evaluated. Shorter closure times indicate increased platelet aggregation. Testing was done in whole blood from antecubital vein samples anticoagulated with 3.8% sodium citrate. Samples were obtained at admission in 135 patients. PFA-100 tests were performed within 30 min after blood sampling, within 1 hour after admission to hospital. The epinephrine-collagen cartridge (EPI-COLL) is sensitive to aspirin and can be used for the detection of aspirin resistance [6]. The ADP-collagen cartridge (ADP-COLL) is only weakly sensitive to aspirin. Aspirin resistance was defined as normal EPI-COLL closure times (<193 sec) based on the 90% central interval in a normal population [6].

### Nucleotide measurements

Nucleotides were measured in a total of 64 patients (16 patients with STEMI, 16 patients with NSTEMI and 32 patients with NCD). ADP was measured with hplc from antecubital vein samples. Sampling was done at admission. 5 ml blood was added to tubes containing citrate and immediately centrifuged for 10 min at 1200 G, 4°C. Platelet contamination was excluded by Burker chamber examination. The plasma was aspirated and mixed with an equal amount of 10% TCA to precipitate all proteins and inactivate ectonucleotideases. After centrifugation the protein free supernatant was frozen at -80°. Samples were sent on dry ice by courier, to Department of Medicine, University of North Carolina, School of Medicine, Chapel Hill, USA for analysis.

Samples were thawed at room temperature and TCA was extracted three times with six volumes of ethyl ether. Ethyl ether was removed by gassing N_2_, and the resulting samples diluted in the corresponding nucleotide assay buffer as indicated below.

#### Luciferin-luciferase assay

This assay has been previously described in detail elsewhere [11]. Typically, extracts were diluted 1:20 in HEPES-buffered Hanks Balanced Salt Solution (HBSS, pH. 7.4) and a 30-μl sample was added to a test tube and the volume adjusted to 300 μl with HPLC-grade H_2_O. The luciferin-luciferase reaction mix (100 μl) was added to tubes with a built-in injector into the light protected chamber of an Auto-Lumat LB953 luminometer. Luminescence was subsequently recorded during 10 seconds and compared against an ATP standard curve performed in parallel. Luminescence was linear with slope of one between 0.1 and 1000 nM ATP.

#### Derivatization of adenosine and adenine nucleotides

We have adopted and slightly modified a derivatization protocol originally described by Levitt and co-workers [12]. Sample extracts were incubated for 30 min at 72°C in the presence of 1.0 M chloroacetaldehyde and 25 mM Na_2_HPO_4 _(pH 4.0) in a 200-μl final volume. Samples were transferred to ice, alkalinized with 50 μl 0.5 M NH_4_HCO_3_, and analyzed by HPLC within 24 h. Identification and quantification of ethenylated species were performed with an automated Waters HPLC apparatus equipped with a fluorescence detector. Derivatized samples were transferred to 0.7 ml plastic shell vials and kept at 4°C in the sample injector rack. A 100 μl sample aliquot was injected into a 250-mm, 10 μm Hamilton PRP-X100 anion exchange column. The mobile phase (2 ml/min, 30 % methanol) developed linearly from 0.250 to 0.275 M NH_4_HCO_3 _(pH 8.5) during the first 8 min, remaining isocratic at 0.275 M NH_4_HCO_3_for additional 4 min. The column was subsequently rinsed for 3 min with 0.425 M NH_4_HCO_3 _in 30% methanol, and re-equilibrated to the initial conditions for 15 min. Elution times (in min) were: ε-ADO, 3.2; ε-AMP, 5.9; ε-ADP, 7.6, and ε-ATP, 9.4.

### Reagents

[^14^C]glucose-1P (300 mCi/mmol) and molecular biology grade ATP and UTP were purchased from Amersham Pharmacia Biotech (Piscataway, NJ). ADP, AMP, and adenosine were from Roche Molecular Biochemicals (Indianapolis, IN). Etheno-adenyl standards were from Sigma (St. Louis, MO). Firefly luciferase and luciferin were purchased from PharMingen International (San Diego, CA). Other chemicals were of the highest purity available.

### Calculation and statistics

Calculations and statistics were performed using the GraphPad Prism 3.02 software. Values are presented as mean ± SD. Statistical significance was accepted when *P *< 0.05 (two-tailed test). For continuous variables Kruskal-Wallis test followed by Dunnett multiple comparisons test was used. Spearman's rank correlation coefficient test was used for regression analysis. Categorical variables were compared using the Fisher exact test.

### Ethics

The Ethics Committee of Lund University approved the project. The study complies with the Declaration of Helsinki. All patients gave written consent to participate in the study.

## Results

### Patient characteristics

The clinical characteristics of the NCD, NSTEMI and STEMI groups are shown in Table 1.

**Table 1:** The clinical characteristics of the NCD (no sign of cardiac disease), NSTEMI (non ST elevation myocardial infarction) and STEMI (ST elevation myocardial infarction) groups. Values are expressed as mean ± SD or numbers.

**Characteristics**	**Controls **n = 67	**NSTEMI **n = 38	**STEMI **n = 30
Age	66 ± 12	72 ± 15	72 ± 13
M/F	46/21	29/9	21/9
Diabetes Mellitus	8	8	4
Prior IHD	29	18	12
Hemoglobine, g/l	135 ± 15	132 ± 15	132 ± 16
Platelet count, ×10^9^/l	224 ± 74	218 ± 51	222 ± 66
Cholesterol mmol/l	-	4,67 ± 0,94	4,60 ± 0,79
Triglycerides mmol/l	-	1,19 ± 0,46	1,28 ± 0,94
BMI	-	24 ± 2,8	27 ± 3,2

### PFA-100

**Figure 1:**
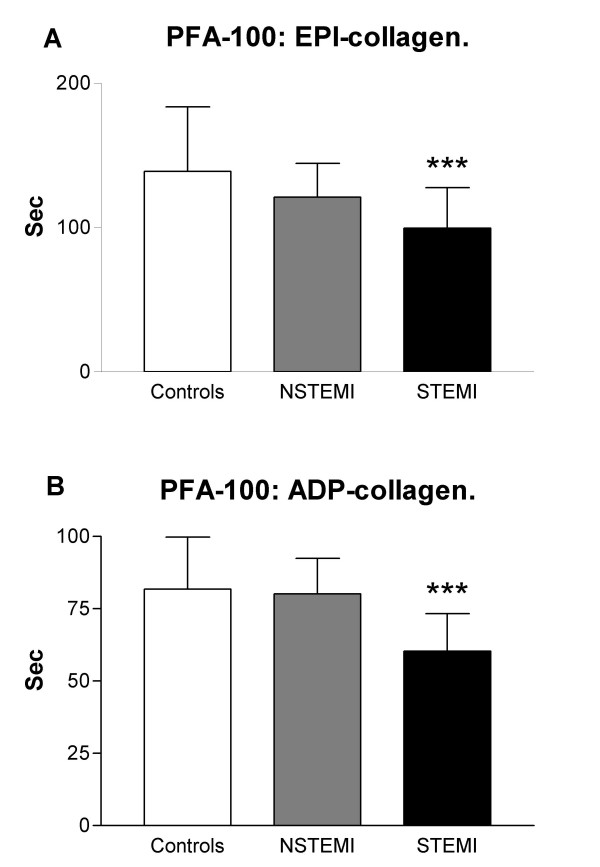
(a) Closure time measurements (epi-collagen) in NCD (no sign of cardiac disease), NSTEMI (non ST elevation myocardial infarction) and STEMI (ST elevation myocardial infarction) groups. (b) Closure time measurements (ADP-collagen) in the NCD, NSTEMI and STEMI groups. *** p < 0.001, compared to NCD. Lower values indicate increased platelet activation.

**Figure 2:**
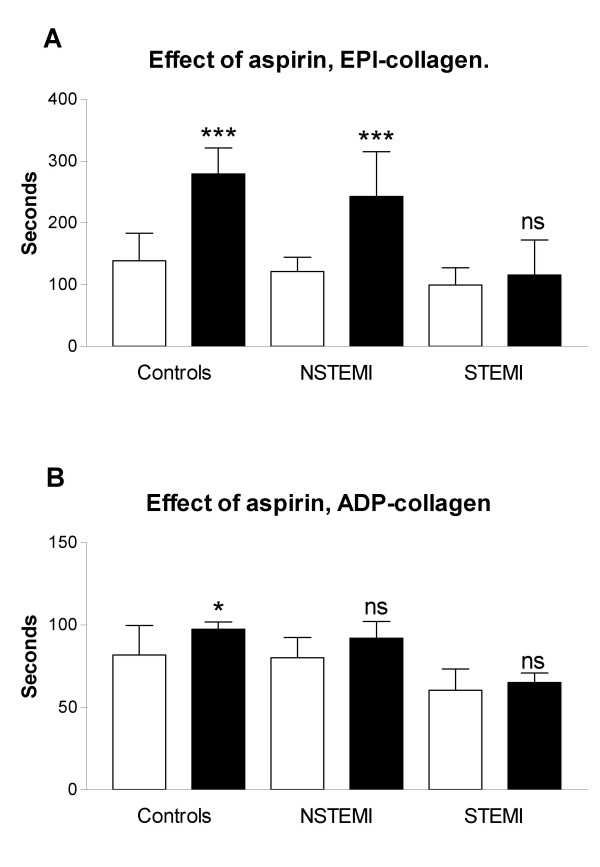
(a) Effect of chronic aspirin treament in the NCD (no sign of cardiac disease), NSTEMI (non ST elevation myocardial infarction) and STEMI (ST elevation myocardial infarction) groups measured as closure time with the EPI-collagen cartridge. (b) Effects of aspirin in the NCD, NSTEMI and STEMI groups measured as closure time with the ADP-collagen cartridge. White bars: no aspirin treatment, black bars: aspirin treated patients. Values are expressed as mean values ± SD, *** p < 0.001, * p < 0.05, n.s. = not significant, compared to NCD. Lower values indicate increased platelet activation.

In patients without aspirin therapy there was an increased platelet activation in the STEMI group compared to NCD in the EPI-coll. NCD: 139 ± 44, NSTEMI: 121 ± 23 (n.s.), STEMI: 99 ± 28 sec (p < 0.001, mean ± SD). (Fig 1a.) These differences were also seen in patients on aspirin. NCD: 280 ± 41, NSTEMI: 243 ± 72 (n.s.), STEMI: 116 ± 56 sec (p < 0.001, Fig 2a). Lower values indicate increased platelet activation. Similar results were observed with ADP-COLL measurements (Fig 1b and 2b).

Chronic aspirin treatment (at least one week before admission) significantly reduced platelet aggregation in NCD and NSTEMI as seen by increased PFA-100 times for EPI-COLL, but only in NCD for ADP-COLL (Fig 2). However, aspirin had no effect in either EPI-COLL or ADP-COLL in patients with STEMI (Fig 2). Aspirin resistance defined as <193 sec in EPI-COLL was 9.7 % in NCD, and increased to 26.0 % in NSTEMI (n.s.) and 83.3 % in STEMI(p < 0,001).

For PFA-100 measurements there were no significant correlation with age, diabetes, hemoglobin, CKMB, troponin T or platelet levels.

### Nucleotide release

**Figure 3:**
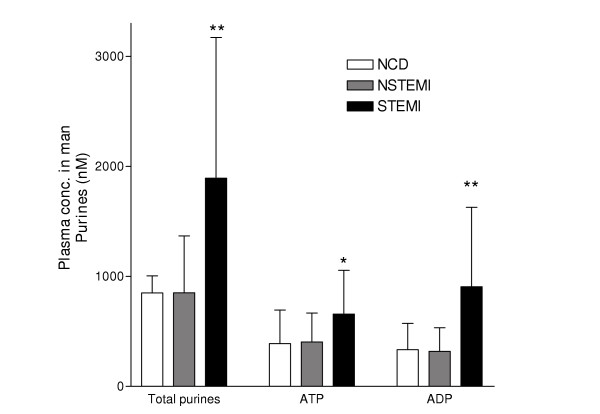
Plasma concentrations of extracellular purines in NCD (no sign of cardiac disease), NSTEMI (non ST elevation myocardial infarction) and STEMI (ST elevation myocardial infarction) groups expressed as mean values ± SD. * p < 0.05, ** p < 0.01, compared to NCD.

**Figure 4:**
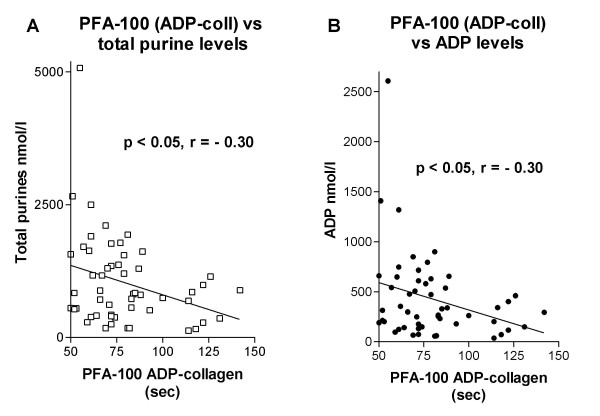
(a) Correlation between extracellular total purine levels and platelet activity measured as closure time with the ADP-COLL cartridge. (b) Correlation between extracellular ADP levels and platelet activity measured as closure time with the ADP-COLL cartridge.

Plasma levels of ADP were markedly increased in STEMI (905 ± 721 nmol/l, p < 0.01), but not in NSTEMI (317 ± 245), compared to NCD (334 ± 271, mean ± SD) (Fig 3). Similar findings were found for other purines (ATP and AMP). ADP levels correlated with increased platelet activity measured with ADP-COLL in the whole material (r = -0.30, p < 0.05, Fig 4a). Similar results were seen for total purines (r = -0.30, p < 0.05, Fig 4b). There was a non-significant trend for EPI-COLL to correlate with both ADP and total purines in patients with aspirin treatment (ADP: r = -0.31, p = 0.09, total purines: r = -0.32, p = 0.09).

Aspirin resistant patients (EPI-COLL < 193 sec) had higher ADP levels compared to aspirin responders (734 ± 807 vs. 282 ± 187 nmol/l, mean ± SD, p < 0.05), and increased levels of total purines (1615 ± 1493 vs. 737 ± 408 nmol/l, mean ± SD, p < 0.05).

For purine measurements there were no significant correlation with age, diabetes, haemoglobin, CKMB or troponin T levels. Platelet contamination was excluded by cell counting and we did not see any correlations between platelet counts and purine levels.

Nucleotide turnover is fast in whole blood due to ectonucleotidases. ATP degradation was evaluated both in samples with endogenous ATP and in samples were ATP had been added. The degradation was rapid in samples where ATP was added with a T 1/2 of 5.2 min. Endogenous ATP levels had a slower degradation rate, with a T 1/2 of approximately 30 min.

Baseline levels of adenosine were markedly lower than those of its nucleotides and barely detectable, most likely due to both rapid uptake into the red blood cells and degradation. This was because we have not included adenosine deaminase and nucleoside transport inhibitors in the perfused solution. The magnitude of changes in adenosine levels could therefore not be studied but this was not the aim of the study.

## Discussion

In agreement with previous studies we found that platelets are activated in acute coronary syndromes [13,14]. Furthermore, for the first time we could demonstrate a rise in systemic levels of ADP and a decreased platelet inhibitory effect of aspirin in patients with STEMI. It is possible that the raised ADP level contributes to the increased platelet activity and the reduced effect of aspirin

There is growing evidence that a significant number of patients do not benefit from therapy with standard doses of aspirin. Aspirin resistance however, is a poorly defined term describing a number of conditions including the inability of aspirin to protect individuals from cardiovascular events, to inhibit platelet aggregation measured with a number of different methods and to inhibit thromboxane A_2 _formation [15]. We found aspirin resistance levels in controls (NCD) of 9.7%, which is in agreement with a previous study that found 9.5% aspirin resistance using PFA-100 [6]. It is also in agreement or even lower than other methods that found frequencies of 9–45%. [6-8]. interestingly, we found increased frequency of aspirin resistance in acute coronary syndromes rising from 9.7% in controls to 26.0 % in NSTEMI (n.s.) and 83.3 % in STEMI (p < 0,001). In fact, we could not see any significant effect of aspirin on platelet aggregation measured with PFA-100 in acute STEMI. Thus, in the situations where the patients need the platelet inhibitory effect of aspirin the most, the aspirin resistance is most frequent

The causative factors of aspirin resistance are still unclear. Altman et al., (2004) described several possible mechanisms behind aspirin resistance and the difficulty to interpret and compare the results of different studies [16]. Both the possibilities of inadequate doses of aspirin and thromboxane independent platelet aggregation mechanisms have been discussed. In some patients aspirin resistance could simply reflect non-compliance with drug therapy. Weber et al., (2002) suggested several possible types of aspirin resistance where one is linked to inability to inhibit thromboxane formation [15]. Another possible mechanism is classified as "pseudo resistance" since aspirin exerted the expected pharmacodynamic effect of inhibiting thromboxane formation but platelet aggregation was not inhibited. Hillarp et al., (2003) found that in a series of 200 aspirin treated patients, none was found to have unblocked cyclooxygenase activity [17]. Aspirin resistance despite blocked cyclooxygenase activity has been suggested to be explained by increased platelet sensivity to ADP and collagen [5,18].

Our data show that aspirin resistance increases in STEMI. This is probably due to a general activation of the platelets, because platelet aggregation was also increased in patients not treated with aspirin. The increased aspirin resistance could be explained by increased activity of important positive feedback systems such as ADP and thromboxane [19], but also by an increase in vW factor that are released from endothelial cells under high shear stress [20].

Several studies have shown sufficient inhibition of platelet aggregation with the 75 mg dose [1,20]. Recently though, a randomized study of 60 patients with stable coronary artery disease showed that the effect of aspirin was dose-dependent and the conclusion was that doses of less than 100 mg of aspirin was less effective inhibiting platelet aggregation than doses greater than 100 mg [22]. This could indicate that higher doses of aspirin could be necessary to inhibit increased activity in the thromboxane positive feedback system, but for the time being there is no clear evidence saying that higher doses of aspirin improve clinical outcome [1].

However, aspirin resistance could also be dependent on increased activity of the ADP positive feedback system. ADP is released by activated platelets, but also in the heart from cardiac myocytes during ischemia, or from endothelial cells, red blood cells and sympathetic nerves [22,23]. Erythrocytes are known to contain large amounts of ADP, which may increase the platelet activity and modulate the effect of aspirin [24]. It is known that nucleotides are released from numerous cells during stress and exercise [23]. The control group in this study was therefore chosen to present pain and stress and it is our belief that this control group is more relevant for comparison than unstressed healthy individuals to prove ischemia induced increases in ADP levels.

For the first time we have demonstrated in man that ADP levels are increased during myocardial infarction. It is possible that increased ADP levels during acute myocardial ischemia could contribute to the increased frequency of aspirin resistance. There are at least two subtypes of ADP receptors on the platelet. The P2Y_12 _receptor is linked via G_i _protein to adenylate cyclase. The P2Y_12 _receptor stimulates platelet aggregation and has a high expression in platelets [25,26]. Clopidogrel blocks the P2Y_12 _receptor irreversibly and the value of this treatment has been established by the CURE and CAPRIE studies. P2Y_1 _is a G_q _protein linked ADP receptor expressed in platelets that mediates shape change [25,26]. P2Y_12 _and thromboxane receptors act via different intracellular second messenger mechanisms, cAMP and inositol triphosphate (IP_3_), respectively. This explains the additive clinical effect of clopidogrel when it is combined with aspirin [3,4]. However, the P2Y_1 _receptor acts via the same second messenger system as thromboxane (IP_3_). Thus, high levels of ADP could replace thromboxane as stimulator of IP_3 _by activation of P2Y_1 _receptors. If ADP contributes to aspirin resistance, it may not be sufficient to block the P2Y_12 _receptor. It is possible that P2Y_1 _antagonists also will be necessary to achieve inhibition of both the important intracellular second messenger systems in the platelet.

Several studies have shown a higher incidence of cardiovascular events in patients reported to be aspirin resistant. Gum et al found that aspirin resistance was associated with an increased risk of death, myocardial infarction or cerebrovascular accident compared to aspirin sensitive patients (24% vs. 10%) [8]. Grotemeyer and colleagues described in 1993 a 40% risk for major events (stroke, myocardial infarction or vascular death) for aspirin resistant patients compared to a 4% risk in the aspirin responder group [7]. Eikelboom et al., (2002) recently reported that elevated urine concentrations of 11-dehydro thromboxane B2 could predict the risk of myocardial infarction [9]. Further prospective studies will be needed to evaluate if ADP levels could be of importance in the identification of patients with increased risk of myocardial infarction in the future. Why did the patients with aspirin resistance have higher ADP levels? It is possible that this was the result of a more pronounced cardiac ischemia. However, ADP levels did not correlate with either CKMB or troponin T levels. Another explanation could be that subgroups of patients are more dependent on the ADP positive feedback system than on the thromboxane system, and therefore more aspirin resistant. Then our finding of increased ADP levels may reflect an increased ADP release from platelets. These patents may benefit more from inhibitors of ADP mediated platelet activation than aspirin.

### Limitations of the study

Patients found to be aspirin resistant were not all confirmed by a second test with the PFA-100. However, this was not possible in the NSTEMI and STEMI groups since they were treated early with clopidogrel, enoxaparin or GPIIb/IIIa-blockers that influence the PFA-100 measurements. ADP measurements are difficult because of the rapid degradation by ectonucleotidases being present predominantly on endothelial cells. We found a half-life of 5.2 min when ATP was added to our blood samples in vitro, however endogenous levels of ATP levels were more stable. The baseline ATP levels were in the micromolar range, which is similar to previously reported levels of circulating ATP in man [27]. Our sampling of venous blood in the antecubital vein is clearly not optimal to detect purine release in the heart. The released purine has passed both the lung and systemic circulation resulting in a degradation chain from ATP, ADP, AMP to adenosine. Adenosine is then rapidly taking up by the red blood cells. Thus the adenosine levels were barely detectable. It is our belief that blood sampling directly from heart veins would have resulted in markedly increased purine levels in the NSTEMI and STEMI groups and probably also better correlations with platelet activity. Another limitation is that the correlation analysis had to be done on the whole material since the subgroups were too small for separate correlation analysis.

## Conclusion

Platelets are activated and aspirin resistance is more frequent in STEMI, probably due to a general activation of platelets. ADP levels are increased in STEMI and correlates with platelet activation. Increased levels of ADP could be one reason for increased platelet activity and aspirin resistance.

## Authors' contributions

CB was the principal investigator for the study, responsible for recruitment of patients, aspirin resistance analysis, study design and wrote the manuscript.

CvH performed the purine analytical assays.

EL performed the purine analytical assays and participated in writing the manuscript.

HÖ participated in study design, helped in recruiting patients and wrote the manuscript.

DE conceived the study, guided throughout the study and wrote the manuscript.

All authors read and approved the final manuscript.
